# Gut microbiota and metabolome signatures in obese and normal-weight patients with colorectal tumors

**DOI:** 10.1016/j.isci.2025.112221

**Published:** 2025-03-13

**Authors:** Marta La Vecchia, Michela Giulia Clavenna, Marika Sculco, Gloria Sala, Denise Marradi, Elettra Barberis, Soni Joseph, Marta Mellai, Nico Pagano, Renzo Boldorini, Barbara Azzimonti, Elisa Bona, Edoardo Pasolli, Flavia Prodam, Carlotta Sacerdote, Daniela Ferrante, Emilia Ghelardi, Marcello Manfredi, Anna Aspesi, Irma Dianzani

**Affiliations:** 1 Department of Health Sciences, Università del Piemonte Orientale, 28100 Novara, Italy; 2 Department of Translational Medicine, Università del Piemonte Orientale, 28100 Novara, Italy; 3 Center for Translational Research on Autoimmune and Allergic Diseases, University of Piemonte Orientale, 28100 Novara, Italy; 4 Department of Sciences and Technological Innovation, Università del Piemonte Orientale, 15121 Alessandria, Italy; 5 Department of Gastroenterology, University Hospital "Maggiore della Carità", 28100 Novara, Italy; 6 Pathology Unit, University Hospital "Maggiore della Carità", 28100 Novara, Italy; 7 Department for Sustainable Development and Ecological Transition, Università del Piemonte Orientale, 13100 Vercelli, Italy; 8 Simple Departmental Structure Research Laboratories - Integrated Activities Research and Innovation Department, Azienda Ospedaliera SS. Antonio e Biagio e Cesare Arrigo, 15121 Alessandria, Italy; 9 Department of Agricultural Sciences, Division of Microbiology, University of Naples Federico II, 80055 Portici, Italy; 10 Task Force on Microbiome Studies, University of Naples Federico II, 80055 Portici, Italy; 11 SCDU Endocrinology, University Hospital "Maggiore della Carità", Department of Translational Medicine, University of Piemonte Orientale, Via Solaroli 17, 28100 Novara, Italy; 12 Department of Translational Research and New Technologies in Medicine and Surgery, University of Pisa, 56127 Pisa, Italy

**Keywords:** Cancer systems biology, Health sciences, Metabolomics, Microbiome

## Abstract

Here, we aim to improve our understanding of various colorectal cancer (CRC) risk factors (obesity, unhealthy diet, and gut microbiota/metabolome alteration), analyzing 120 patients with colon polyps, divided in normal-weight (NW) or overweight/obese (OB). Dietary habits data (validated EPIC questionnaires) revealed a higher consumption of processed meat among OB vs. NW patients. Both mucosa-associated microbiota (MAM) on polyps and lumen-associated microbiota (LAM) analyses uncovered distinct bacterial signatures in the two groups. Importantly, we found an enrichment of the pathogenic species *Finegoldia magna* in MAM of OB patients, regardless of their polyp stage. We observed distinct mucosal-associated metabolome signatures, with OB patients showing increased pyroglutamic acid and reduced niacin levels, and performed microbiota-metabolome integrated analysis. These findings support a model where different risk factors may contribute to tumorigenesis in OB vs. NW patients, highlighting the potential impact of processed meat consumption and *F. magna* on CRC development among OB patients.

## Highlights

• Tumor-adherent microbiota and metabolome vary between OB and NW patients
• The pathobiont *F. magna* is enriched on colorectal polyps of OB patients
• Obesity, diet, and gut microbiota cooperate in CRC tumorigenesis in OB patients

## Graphical abstract

**Figure:**
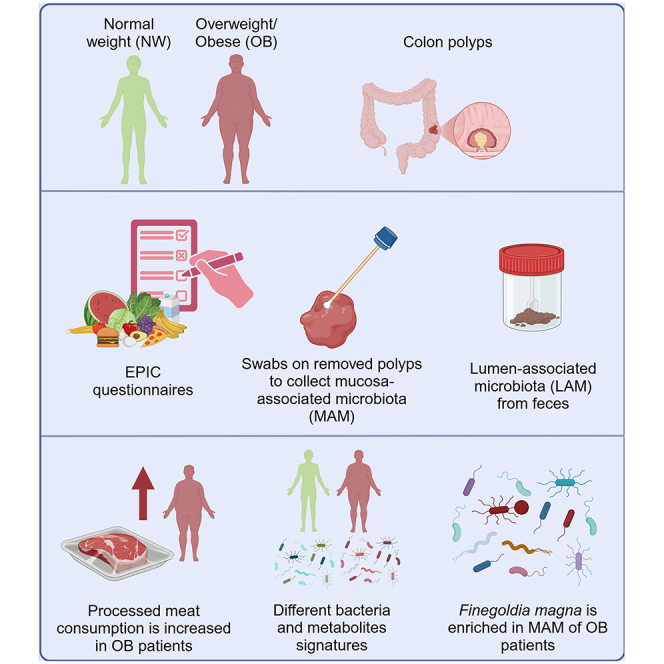

## Introduction

Trillions of microorganisms, collectively referred to as the gut microbiota, colonize human intestine and significantly influence physiology and pathology.^1^ The gut microbiota plays a pivotal role in modulating the maturation and function of immune cells. Indeed, several diseases, characterized by inflammation and autoimmunity, have been recently linked to compositional and functional alterations in the gut microbiota, known as intestinal dysbiosis.^2^

The involvement of the intestinal microbiota in colon carcinogenesis, either directly or indirectly through the production of metabolites able to increase enterocyte proliferation and support opportunistic pathogens,^3^ is now widely recognized. Interestingly, also gut inflammation and obesity, both established risk factors for colorectal cancer (CRC), have been associated with gut dysbiosis,^4^ suggesting a biological link among these conditions.

Specifically, emerging evidence has shown that increased body mass index (BMI) correlated with the presence of colorectal polyps and CRC development.^4^^,^^5^ Proposed pathogenic mechanisms include: (1) the activation of insulin and insulin-like growth factor receptor pathways and the ensuing induction of proliferation and inhibition of apoptosis, (2) the proinflammatory effect of secondary bile on a high-fat diet, and (3) systemic inflammation.^4^

The clear distinction in gut microbiota between lean and obese individuals underscores the significant role that these microorganisms play in weight regulation. This is particularly evidenced by experiments where transferring gut bacteria from fat to lean individuals results in weight gain in the former, and vice versa.^6^ In this regard, several microbial species have been identified as either exacerbating or preventing obesity. For example, chronic low-grade inflammation, a hallmark of obesity,^7^ leads to intestinal dysbiosis, creating an environment conducive to the growth of bacteria like *Enterobacteriaceae*.^8^ These bacteria deplete oxygen in the gut, paving the way for the colonization of other bacteria, such as *Clostridium* and *Bacteroides*, which together with *Enterobacteriaceae,* may trigger the onset of CRC.^9^^,^^10^

The typical diet of obese individuals, rich in red and processed meats, sugary drinks, refined grains, and saturated fats, with low intake of fruits, vegetables, whole grains, fish, nuts, and seeds,^11^ is a classic example of the Western dietary pattern. This diet, by promoting excessive energy intake, leads to fat accumulation and, consequently, chronic inflammation. Western diet is also associated with an increased risk of CRC and its recurrence.^12^ Consuming large amounts of red and processed meats further disrupts the gut balance, favoring the proliferation of specific bacteria, such as *Bacteroides* and *Erysipelotrichaceae*,^13^^,^^14^ candidate driver and passenger genera, respectively involved in CRC initiation and progression.^9^

Based on the aforementioned evidence, the aim of this study was to improve our understanding of the various risk factors for CRC (i.e., obesity, diet, gut microbiota, and metabolome) and their status in obese vs. normal-weight CRC patients. To this end, we collected anthropometric parameters, dietary habits—through a validated EPIC questionnaire^15^—and samples of mucosa-associated and lumen-associated microbiota (MAM and LAM, respectively), as well as mucosal and fecal metabolites from a cohort of 120 patients carrying colorectal polyps.

## Results

A total of 120 incidental patients (71 males and 49 females) carrying polyps larger than 1 cm were enrolled before undergoing colonoscopy at the Gastroenterology Unit of the University Hospital Maggiore della Carità in Novara, Italy. Their clinical characteristics are listed in Table 1. The analysis revealed that there were no statistically significant differences between normal-weight (NW) and overweight/obese (OB) patients in terms of sex, age, polyp histology, polyp grade of dysplasia, polyp localization, polyp dimension, smoking and physical activity habits, previous gastrointestinal conditions and comorbidities. OB patients showed a statistically significant lower educational level than NW patients (*p* = 0.01). The flow chart of the analysis performed on the study population is reported in Figure S1.

**Table 1:** Clinical features of our study population

	Patients (*N* = 120)	Normal weight (*N* = 75)	Obese (*N* = 45)	*p*-value
Sex				
Male (%)	70 (58.3%)	46 (61.3%)	24 (53.3%)	0.39 (chi-squared)
Female (%)	50 (41.7%)	29 (38.7%)	21 (46.7%)	
Age				
Mean (SD)	62 (9.7)	62 (10.0)	62 (9.3)	0.88 (unpaired t-test)
Polyp histology				
Tubular, Villous, Tubulo-villous adenomas	93 (77.5%)	53 (70.7%)	40 (88.9%)	0.07 (Fisher’s exact test)
Adenocarcinoma	11 (9.2%)	9 (12%)	2 (4.4%)	
Hyperplastic, Serrated, Sessile-serrated	16 (13,3%)	13 (17.3%)	3 (6.7%)	
Polyp grade of dysplasia				
Low-grade group (%)^a^	65 (54.2%)	37 (49.30%)	28 (62.2%)	0.17 (chi-squared)
High-grade group (%)^a^	55 (45.8%)	38 (50.70%)	17 (37.8%)	
Polyp localization				
Distal (%)	75 (62.5%)	45 (60.0%)	30 (66.7%)	0.46 (chi-squared)
Proximal (%)	45 (37.5%)	30 (40.0%)	15 (33.3%)	
Polyp dimension				
Mean in mm (SD)	18.27 (10.3)	18.36 (11.0)	18.15 (9.0)	0.91 (unpaired t-test)
BMI				
Mean in Kg/m^2^ (SD)	26.1 (5.3)	23.4 (2.8)	30.5 (5.6)	<0.0001 (unpaired t-test)
WC				
Mean in cm (SD)	93.9 (14.6)	85.8 (10.2)	106.8 (11.1)	<0.0001 (unpaired t-test)
Smoking				
Yes (%)	26 (21.7%)	15 (20.0%)	11 (24.4%)	0.69 (chi-squared)
Only in the past (%)	54 (45.0%)	33 (44.0%)	21 (46.7%)	
Never (%)	40 (33.3%)	27 (36.0%)	13 (28.9%)	
Physical activity index				
Inactive (%)	31 (25.8%)	15 (20.0%)	16 (35.5%)	0.32 (Fisher’s exact test)
Moderately inactive (%)	51 (42.5%)	35 (46.7%)	16 (35.5%)	
Moderately active (%)	12 (10.0%)	8 (10.7%)	4 (9.0%)	
Active (%)	25 (20.8%)	16 (21.3%)	9 (20.0%)	
Unavailable (%)	1 (0.8%)	1 (1.3%)	0 (0.0%)	
Previous gastrointestinal conditions				
Diverticulitis (%)	39 (32.5%)	21 (28.0%)	18 (40.0%)	0.94 (Fisher’s exact test)
Previous polyp occurrence (%)	12 (10.0%)	6 (8.0%)	6 (13.3%)	
IBD (%)	1 (0.8%)	0 (0.0%)	1 (2.2%)	
Previous cholecystectomy (%)	5 (4.2%)	3 (4.0%)	2 (4.4%)	
Slight mucosal inflammation (%)	1 (0.8%)	1 (1.3%)	0 (0.0%)	
Comorbidities (heart attack, stroke and/or diabetes)				
Yes (%)	15 (12.5%)	6 (8.0%)	9 (20.0%)	0.054 (chi-squared)
No (%)	105 (87.5%)	69 (92.0%)	36 (80.0%)	
Educational level				
Primary school (%)	28 (23.3%)	14 (18.7%)	14 (31.1%)	0.01 (Fisher’s exact test)
Junior high school (%)	32 (26.7%)	14 (18.7%)	18 (40.0%)	
High school (%)	32 (26.7%)	25 (33.3%)	7 (15.6%)	
Vocational school (%)	15 (12.5%)	11 (14.7%)	4 (8.9%)	
University degree (%)	13 (10.8%)	11 (14.7%)	2 (4.4%)	

SD, standard deviation; BMI, body mass index; WC, waist circumference.

a “Low-grade” group: hyperplastic polyps, serrated polyps without dysplasia, or low-grade dysplasia adenomas. “High-grade” group: high-grade dysplasia adenomas or high-grade dysplasia serrated polyps or adenocarcinomas.

In the questionnaire, 79 patients reported that they did not take antibiotics or probiotics in the six months prior to colonoscopy, whereas 6 additional patients reported that they did not take antibiotics or probiotics in the four weeks before colonoscopy. The remaining 35 patients did not provide a reliable information.

### LAM and MAM composition

MAM was collected brushing the adenoma surface after polyp removal using an e-NAT swab, which allows preserving the nucleic acids until extraction, while LAM was obtained from fecal samples.^16^ All samples were analyzed using 16S rRNA sequencing, revealing average reads of 34,973.53 (±25,525.67 SD) for MAM and 56,761.96 (±22,519.59 SD) for LAM. Statistical analysis using MicrobAT, after applying a low-count filter to exclude taxa with fewer than 4 reads, identified 257 taxa in MAM and 287 in LAM.

### MAM signatures differentiate between normal-weight and obese patients

**Figure 1:**
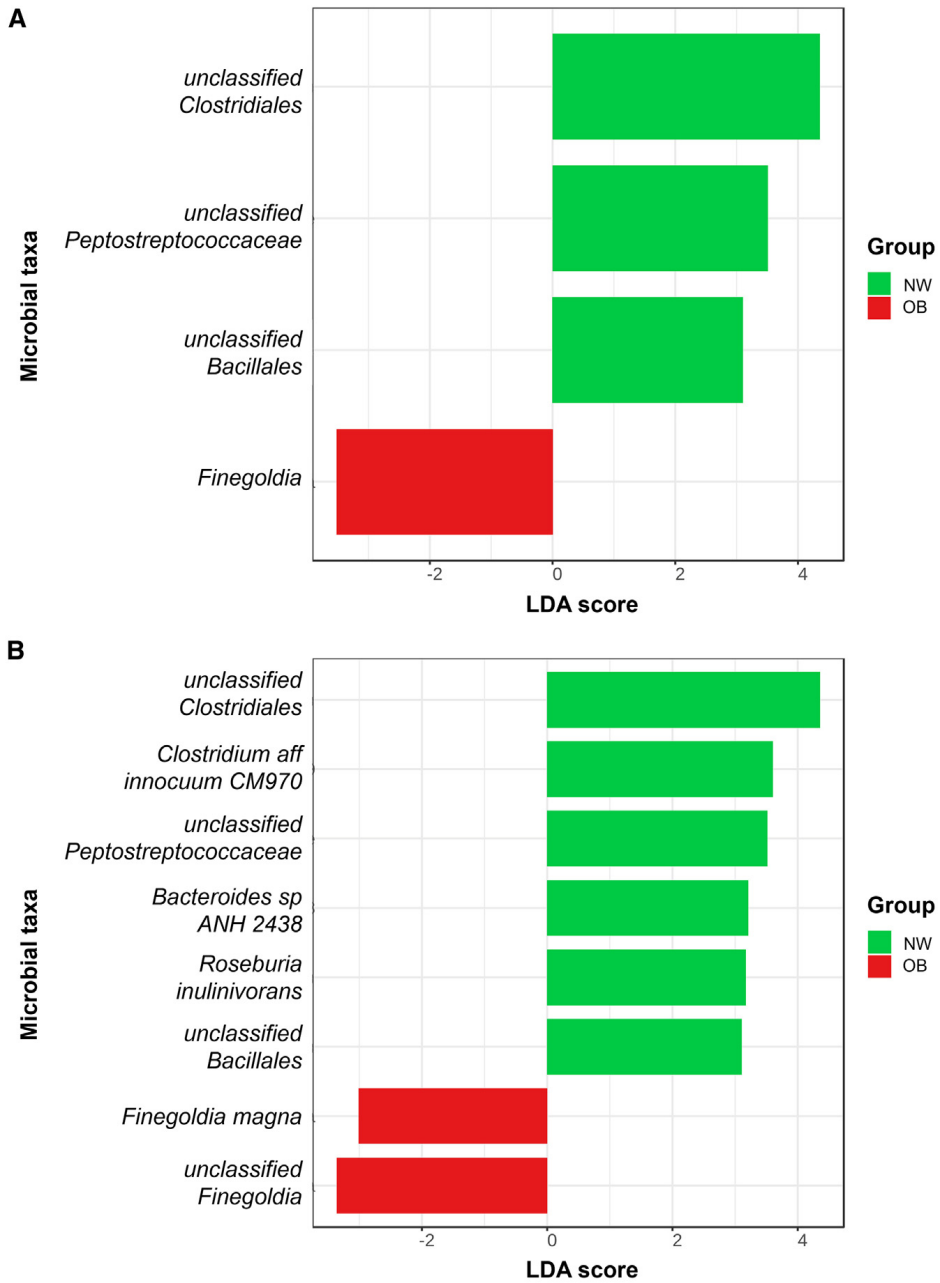
Mucosa-associated microbiota signatures Bacterial genera (A) and species (B) enriched in MAM from normal-weight (NW) and obese (OB) patients. Genera and species enriched in NW patients are highlighted in green (LDA score >2), whereas those prevalent in OB patients are shown in red (LDA score < −2). Data are represented as bars, ranking discriminative features found by LEfSe analysis (*p* < 0.05) according to their LDA score.

Patients with colorectal polyps were divided based on their BMI and waist circumference (WC) into NW (75 subjects) and OB (45 subjects). No statistically significant difference in α-diversity and β-diversity was observed between the two groups (Figure S2). LEfSe analysis of the two groups revealed an enrichment of the genus *Finegoldia*, in particular the species *Finegoldia magna*, in OB vs. NW patients (Figure 1).

To determine whether the enrichment of *F. magna* observed in OB patients correlated with specific carcinogenesis pathways or stages, we refined our analysis by excluding patients with hyperplastic polyps, serrated polyps, and adenocarcinomas. We found that *F. magna* was associated with obesity in patients with tubular, tubulovillous, or villous polyps (*n* = 93) (Figure S3). This result demonstrates that *F. magna* is not specifically linked to adenocarcinomas, hyperplastic or serrated polyps. Moreover, when categorizing OB patients according to the grade of dysplasia of their polyps (low-grade vs. high-grade), we did not observe any significant difference in *F. magna* enrichment (Figure S4), suggesting that its correlation with obesity persists regardless of the polyp stage.

To ascertain whether other factors would influence *F. magna* abundance, we evaluated subgroups of patients according to polyp localization and adherence index to the Mediterranean diet (Tables 1 and S1). In particular, we found an enrichment of *F. magna* in the distal compared with the proximal group, when obesity was not considered, and an enrichment of *F. magna* and *unclassified Finegoldia* in OB patients with distal polyps compared to NW patients with distal polyps (Table S1). *F. magna* and *unclassified Finegoldia* were not enriched in OB patients in any tertile of adherence index to the Mediterranean diet (Table S1). This may depend on the small numerosity of the subgroups.

### LAM signatures differentiate between normal-weight and obese patients

LDA LEfSe analysis detected distinct enriched bacteria in the LAM of both NW and OB patients. Specifically, OB patients showed a higher abundance of the genera *Clostridium XIVb, Butyricicoccus, Phascolarctobacterium, Ruminococcus2, Collinsella,* and *Erysipelotrichaceae incertae sedis*. In contrast, NW patients displayed an enrichment of *unclassified Veillonellaceae* (Figure S5). This differential bacterial presence indicates that patients with different body weights display distinct gut microbiota compositions. When we refined our analysis by excluding patients with hyperplastic polyps, serrated polyps, and adenocarcinomas, we found that *Clostridium XIVb, Butyricicoccus, Erysipelotrichaceae incertae sedis, Ruminococcus2* were still enriched in OB patients (Figure S6). Moreover, when categorizing OB patients according to the grade of dysplasia of their polyps (low-grade vs. high-grade), we observed that none of the bacteria found enriched in LAM of OB patients when compared to NW (Figure S6) vary with the dysplasia grade of the colon polyps of OB patients (Figure S7), suggesting that their correlation with obesity persists regardless of the polyp stage.

### Dietary habits

Dietary habits, gathered through a validated EPIC questionnaire^15^ for 119 out of 120 patients, were assessed for intake of red meat, processed meat, fruits and vegetables, total fiber, and total lipids with and without adjustment for total energy intake. This assessment evidenced that consumption of processed meat was the only dietary pattern to show a statistically significant difference between NW and OB patients (Table 2). Fiber consumption was higher in NW patients, but this difference was not statistically significant (Table 2). Energy intake was not significantly different between the two groups (*p* = 0.25) (Table 2). Noteworthy, the adherence index to the Mediterranean diet was not statistically different between the two groups (*p* = 0.346). In particular, dividing the patients in tertiles according to the adherence index to the Mediterranean diet, 31.1% of normal-weight patients are in the 1st tertile, 51.3% in the 2nd tertile, and 17.6% in the 3rd tertile, whereas 40% of obese patients are in the 1st tertile, 51.1% in the 2nd tertile and 8.9% in the 3rd tertile.

**Table 2:** Average food consumption of specific foods in NW and OB patients

	Normal weight (*n* = 74) median (IQR)	Obese (*n* = 45) median (IQR)	*p*-value
Dressing dips	0.6 (0.1–1.9)	0.7 (0.3–1.6)	0.52
Dressing dips (energy adjusted)	0.6 (0.1–1.9)	0.7 (0.3–1.6)	0.49
Red meat	51.0 (30.2–82.6)	72.1 (34.1–96.2)	0.13
Red meat (energy adjusted)	51.3 (29.8–82.7)	73.5 (34.4–98.1)	0.12
Processed meat	11.4 (6.0–29.6)	22.6 (11.6–38.7)	**0.0095**^a^
Processed meat (energy adjusted)	11.6 (6.0–29.6)	22.6 (11.6–38.2)	**0.0083**^a^
Fruit and vegetables	436.2 (335.9–567.5)	441.9 (292.3–511.0)	0.96
Fruit and vegetables (energy adjusted)	436.7 (333.9–568.1)	441.9 (295.2–511.0)	0.99
Fiber	19.8 (14.8–24.9)	18.0 (12.7–20.7)	0.058
Fiber (energy adjusted)	19.8 (14.8–24.9)	18.0 (13.0–20.7)	0.056
Lipids	73.8 (58.9–96.1)	76.4 (50.8–88.9)	0.36
Lipids (energy adjusted)	73.8 (59.3–96.2)	76.5 (51.9–88.9)	0.37
Energy (kcal)	1822.99 (1545.32–2284.61)	1779.55 (1299.31–2249.89)	0.25

IQR, interquartile range

a Statistically significant differences (*p* < 0.05) are highlighted in bold.

Patients carrying *Finegoldia* on their polyps, both NW (27) and OB (26) showed a significantly higher intake of processed meat compared with those without this bacterium (*p* = 0.001). However, Spearman correlation indexes indicated that the levels of processed meat consumption and the levels of *Finegoldia* on colorectal polyps were independent variables, with Spearman’ rho for the total patient population with *Finegoldia* at 0.1237, for NW patients at 0.1905, and for OB patients at 0.0605.

We also performed Spearman correlation analysis between *Finegoldia* and the consumption of other classes of food and no correlation was found (Spearman’s rho for the total patient population with *Finegoldia* and dressing dips = 0.0304; Spearman’s rho red meat = 0.0876; Spearman’s rho fruit and vegetables = 0.1297; Spearman’s rho fibers = −0.0006; Spearman’s rho lipids = −0.1051).

Moreover, when patients were classified according to the grade of dysplasia of their polyps, no statistically significant differences in any food categories analyzed were found between low-grade or high-grade groups (Table 3).

**Table 3:** Average food consumption of specific foods in patients classified according to the grade of dysplasia of their polyps (low-grade vs. high-grade)

	Low-grade group (*n* = 64) median (IQR)	High-grade group (*n* = 55) median (IQR)	*p*-value
Dressing dips	0.8 (0.2–1.9)	0.4 (0.0–1.6)	0.07
Dressing dips (energy adjusted)	0.8 (0.2–1.9)	0.4 (0.0–1.6)	0.07
Red meat	51.2 (29.7–94.0)	65.6 (39.3–78.9)	0.80
Red meat (energy adjusted)	51.8 (29.7–93.4)	67.3 (39.3–78.7)	0.78
Processed meat	14.3 (7.6–34.1)	16.8 (7.4–31.7)	0.83
Processed meat (energy adjusted)	14.4 (7.6–33.8)	16.8 (7.6–31.7)	0.85
Fruit and vegetables	441.1 (330.7–515.3)	437.4 (308.3–581.7)	0.86
Fruit and vegetables (energy adjusted)	443.0 (330.8–515.5)	437.8 (308.6–581.3)	0.84
Fiber	18.2 (13.9–21.6)	19.6 (12.7–24.5)	0.47
Fiber (energy adjusted)	18.2 (14.0–21.6)	19.6 (13.0–24.4)	0.47
Lipids	76.0 (57.3–87.9)	74.3 (55.2–97.5)	0.70
Lipids (energy adjusted)	76.1 (58.1–87.9)	74.3 (55.8–97.4)	0.72

IQR, interquartile range.

### Mucosa-associated metabolome signatures differentiate between normal-weight and obese patients

**Figure 2:**
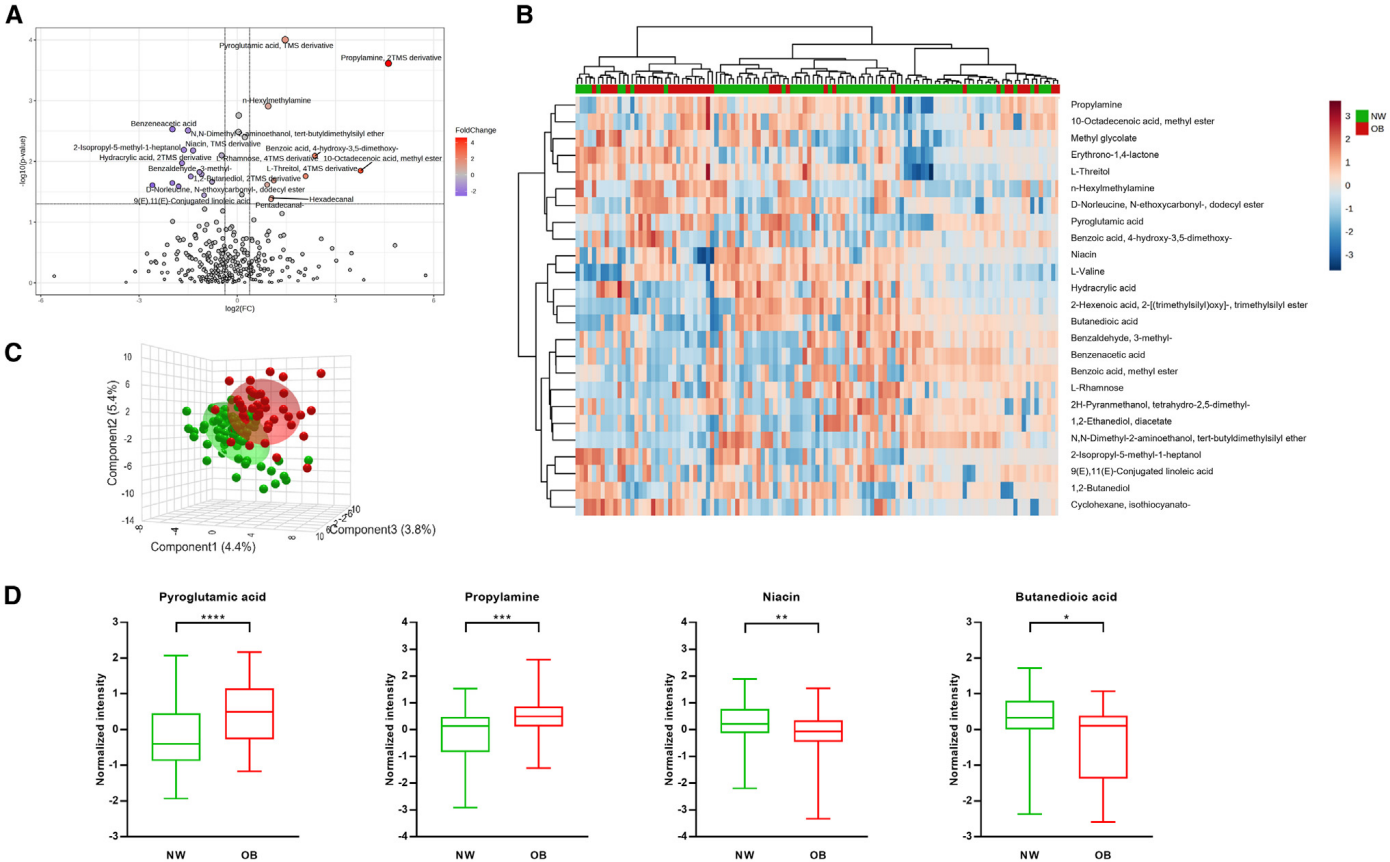
Mucosa-associated metabolome profile (A) Volcano plot reporting upregulated (red dots) and down regulated (blue dots) mucosa-associated molecules in obese (OB) patients. Each dot represents one metabolite. The x axis represents log_2_(FC) of abundances of each metabolite and the y axis represents the statistical significance (-log_10_(*p*-value)). (B) Hierarchical clustering heatmap showing different metabolite distributions between normal-weight (NW) (green) and OB (red) patients. All the metabolites listed show a statistically significant difference between NW and OB groups (*p* < 0.05). Higher concentrations are reported in red, while low levels are in blue (auto-scaled data). (C) Partial least square discriminant analysis (PLS-DA) showing different mucosa-associated metabolite distribution between NW (green) and OB (red) patients. Each dot represents one patient. (D) Boxplots of some of the most statistically significant mucosa-associated molecules discriminating NW (green) and OB (red) patients (∗∗∗∗: *p* < 0.0001; ∗∗∗: *p* < 0.001; ∗∗: *p* < 0.01; ∗: *p* < 0.05). Data are represented as metabolite abundances. NW: normal-weight, OB: obese patients.

Mucosa-associated metabolome analysis on 115 patients allowed the identification and quantification of 327 mucosa associated small molecules. Through statistical analysis, distinctive metabolite profiles were delineated for NW and OB patients with dysplastic polyps, employing an FC-based criterion (FC > 1.3 indicating enrichment in OB patients; FC < 0.769 indicating depletion; *p* < 0.05). This analysis, visualized in a volcano plot (Figure 2A), highlighted the presence of 10 molecules at higher concentration (red dots) and 14 at reduced levels in OB patients, indicating significant differences between the two groups. In this regard, the hierarchical clustering heatmap (Figure 2B) shows the distribution of the top 25 mucosa-associated metabolites statistically different (t-test) between NW vs. OB patients. Furthermore, the partial least square discriminant analysis (PLS-DA) plot (Figure 2C) reveals the presence of a specific metabolome fingerprint associated with OB patients with dysplastic polyps. In particular, in these patients, we found a higher concentration of pyroglutamic acid (FC = 3.04; *p* < 0.01) and propylamine (FC = 15.64; *p* < 0.01), whereas in NW patients we observed a higher concentration of niacin (FC = 0.44; *p* < 0.01) and butanedioic acid (FC = 0.27; *p* < 0.05) (Figure 2D).

### Luminal metabolome signatures differentiate between normal-weight and obese patients

**Figure 3:**
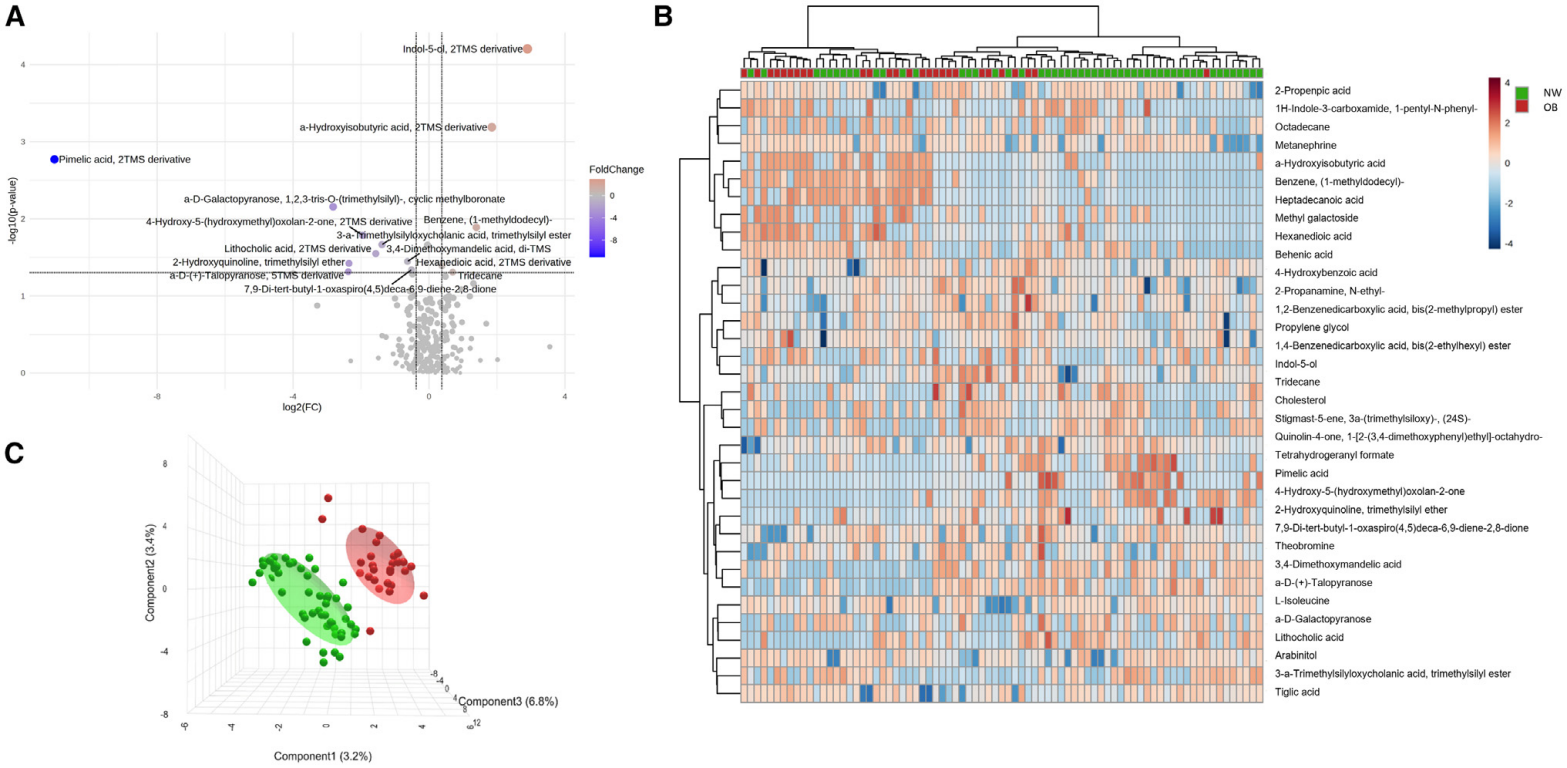
Luminal metabolome profile (A) Volcano plot reporting upregulated (red dots) and down regulated (blue dots) luminal associated molecules in obese (OB) patients. Each dot represents one metabolite. The x axis represents log_2_(FC) of abundances of each metabolite and the y axis represents the statistical significance (-log_10_(*p*-value)). (B) Hierarchical clustering heatmap showing different metabolite distributions between normal-weight (NW) (green) and OB (red) patients. All the metabolites listed show a statistically significant difference between NW and OB groups (*p* < 0.05). Higher concentrations are reported in red, while lower levels are in blue (auto-scaled data). (C) Partial least square discriminant analysis (PLS-DA) showing different luminal-associated metabolite distribution between NW (green) and OB (red) patients. Each dot represents one patient. NW, normal-weight, OB, obese patients.

The metabolome of fecal samples from 79 out of the 120 patients was analyzed, enabling the identification and quantification of 273 small molecules, of which four were enriched and ten were less concentrated in obese patients, as judged by the aforementioned FC criterion (FC > 1.3 enrichment; FC < 0.769 depletion; *p* < 0.05). This was visually represented in a volcano plot (Figure 3A). Hierarchical clustering and PLS-DA analysis further differentiated the metabolic profiles of NW vs. OB patients (Figures 3B and 3C). PLS-DA of fecal samples (Figure 3C) better discriminate between NW and OB patients than PLS-DA of mucosa-associated samples (Figure 2C). In particular, higher levels of indol-5-ol (FC = 7.44; *p* < 0.01) and hexanedioic acid (FC = 1.31; *p* < 0.05) were detected in fecal samples from OB patients, whereas in a higher concentration of pimelic acid (FC = 0.0005; *p* < 0.01) was found in NW patients (Figure S8).

### Integrated mucosa-associated microbiota-metabolome analysis

**Figure 4:**
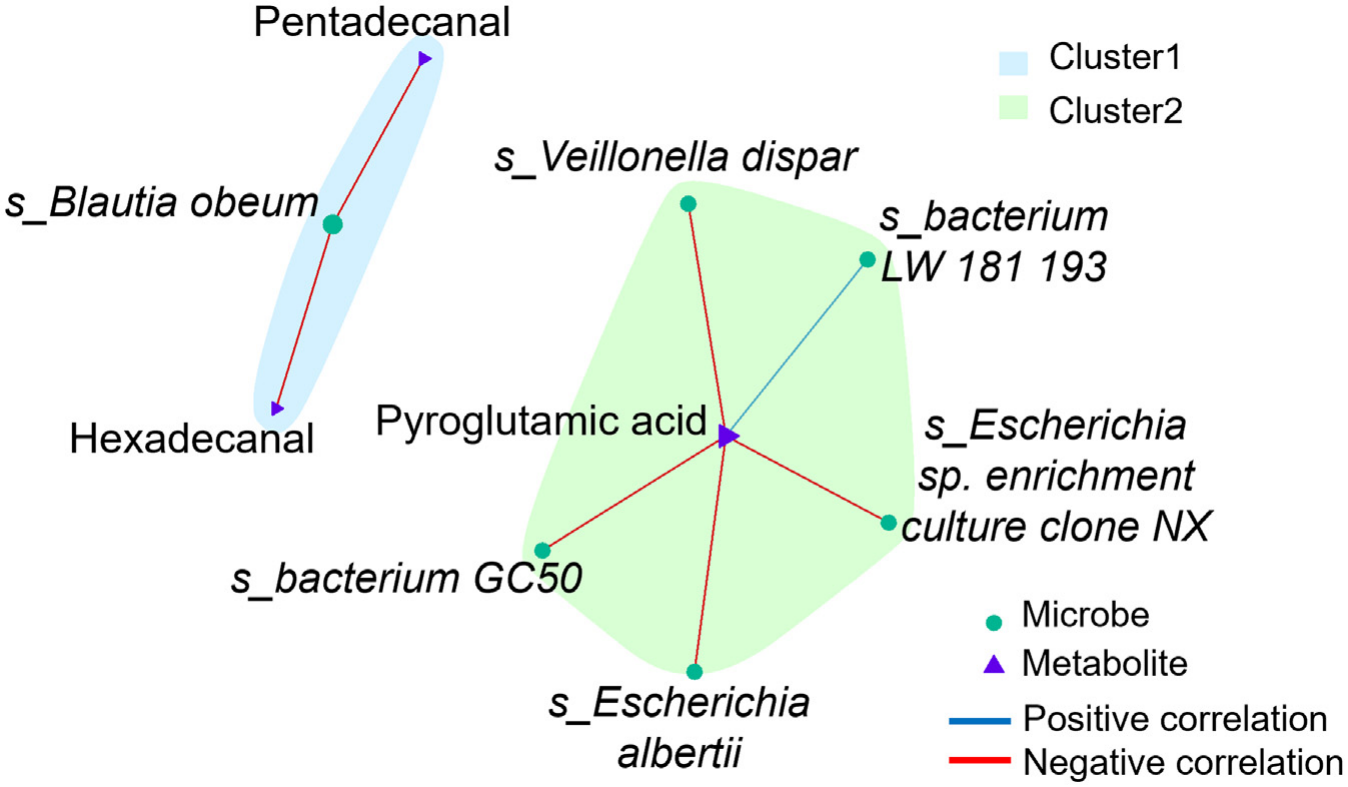
Interaction networks between specific microbes and metabolites at the species level Nodes in the network represent key metabolites or microbes identified using 3MCor software, with solid lines indicating positive (blue) or negative (red) correlations.

To further investigate the relationship between mucosa-associated bacteria and mucosa-associated metabolites, we integrated microbiota and metabolome data using network correlation analysis. For this analysis microbial species abundances and modulated metabolites were used. This approach revealed that pyroglutamic acid, which is more abundant in OB patients compared to NW (Figure 2D), showed a negative correlation with *Veillonella dispar* and *Escherichia albertii* (Figure 4), suggesting a complex interaction between specific bacteria and metabolites, especially in the context of obesity.

An advanced correlation strategy through hierarchical clustering analysis and 3MCor software uncovered significant associations between microbial genera and metabolites (marked with an asterisk in Figure S9A). Using principal component analysis, metabolic and microbial variables were clustered into different modules, which were then correlated (Spearman *p* < 0.05). At the genus level, the Mic4 cluster, which includes *Microbacterium, Fusobacterium, Prevotella, Odoribacter, Phyllobacterium, Lachnospiracea incertae sedis, Clostridium XVII, Pelomonas, Alistipes, Blautia,* and *Escherichia Shigella*, significantly correlated with the Meta6 cluster, comprising hydracrylic acid, L-rhamnose, butanedioic acid, benzaldehyde, benzoic acid, 2H-pyranmethanol, linoleic acid, hexadecanal, pentadecanal, and benzeneacetic acid (Figures S9A−S9C). However, at the species level, no statistically significant associations between microbiota and metabolome clusters were identified (Figure S9D).

## Discussion

In this study, we sought to determine whether obesity and normal-weight conditions in patients with colorectal polyps led to different risk factors for CRC. For this purpose, we assessed obesity, diet, gut microbiota, metabolome and colorectal polyp development in a cohort of 120 patients with colorectal polyps categorizing them into NW and OB groups according to their BMI and WC.

We found that OB patients consumed more processed meat than their NW counterparts (*p* = 0.0095). Furthermore, while fiber consumption was generally higher in NW patients, this difference was not statistically significant (*p* = 0.0588) (Table 2). Processed meat, which is mostly derived from pork or beef meat treated to increase preservation and change flavor, such as bacon, ham and sausages, poses a much higher CRC risk, potentially up to ten times higher than that of fresh red meat.^17^^,^^18^ Thus, our findings align with the notion that the increased CRC risk in OB patients is partly due to their nutritional habits.

Our analyses on both MAM and LAM unveil different signatures in NW vs. OB patients with colon polyps (Figures 1 and S5), underscoring differences in microbiota composition. This is in keeping with our prior research, which demonstrated that MAM, harvested using a novel histology-preserving approach, and LAM differ in composition.^16^^,^^19^ Furthermore, our analysis suggests that the fecal metabolome better discriminates between NW and OB patients than the mucosa-associated metabolome, suggesting that fecal samples more accurately reflect dietary influences, while mucosa-associated samples are more representative of the microenvironment surrounding the polyps (Figures 2C and 3C).

We evaluated both MAM and LAM because the first represent the bacteria that directly interact with the intestinal mucosa, whereas the second represent the broader gut ecosystem and may include potential biomarkers, helpful for the clinic.^16^

Our MAM analysis shows a significant enrichment of the *Finegoldia* genus and *F. magna* species in OB vs. NW patients (Figure 1), a finding not paralleled in LAM. Since we did not find *F. magna* enriched in LAM, it will not be possible to easily evaluate the effects of its modulation in the gut microbiota of OB patients. This is in good agreement with research showing reduced presence of *F. magna* in stool samples compared to rectal swabs^20^ and its greater abundance in mucosal over fecal niches.^21^

*F. magna* (formerly *Peptostreptococcus magnus*) is an opportunistic Gram-positive anaerobic coccus known for colonizing the skin and mucosal membranes, including those in the gastrointestinal tract.^22^ It is recognized for its significant pathogenic potential due to its virulence factors.^23^ Over 90% of *F. magna* isolates express the *F. magna* adhesion factor (FAF), which facilitates bacterial adhesion to the host membrane.^24^ FAF, together with subtilisin-like protease of *F. magna* (SufA) and superantigen protein L, protects the bacteria from host defenses.^23^ In particular, SufA proteolytically inactivates antimicrobial peptides and inhibits fibrin polymerization, favoring bacterial spread to deeper tissues,^25^ while protein L can induce the release of pro-inflammatory mediators.^26^

In Crohn’s disease, an increase in pro-inflammatory pathogens, including *Finegoldia*, in feces can predict clinical relapse.^27^ Moreover, *Finegoldia* spp. have also been found in patients with colorectal adenomas, but not in healthy controls,^28^ suggesting its involvement in the early stages of CRC. Lastly, *Finegoldia* has been associated with *APC*-mutated colorectal tumors,^29^ which typically originate from the adenoma-carcinoma sequence rather than the serrated pathway.^30^ This points to the potential role of *F. magna* in CRC development in OB patients by neutralizing host defenses and promoting inflammation.

Our study also indicates that *F. magna* enrichment is independent of polyp histology, a pattern that remains consistent even after excluding patients with specific types of polyps, such as adenocarcinomas and hyperplastic or serrated polyps (Figure S3). The fact that *F. magna* enrichment does not depend on the dysplasia grade of colon polyps (Figure S4) points to its potential involvement in CRC initiation and progression. Moreover, we found *F. magna* enriched on distal polyps compared to proximal ones, when obesity was not considered. This is in agreement with the observation that a higher abundance of *F. magna* is found in distal vs. proximal colon mucosa.^31^ We also found *Finegoldia magna* and *unclassified Finegoldia* enriched in OB patients with distal polyps compared to NW patients with distal polyps. This suggests that *F. magna* enrichment may be related both to obesity and distal polyp localization.

Another important observation of our study is that we did not find a significant link between the amount of processed meat consumption and the levels of *Finegoldia* in MAM. This suggests that *Finegoldia* enrichment on OB patients’ polyps is more closely related to obesity itself rather than dietary factors, and that processed meat intake may influence a broader bacterial community rather than single species like *F. magna*.

In analyzing mucosa-associated metabolome of 115 patients—samples of five patients were unavailable—we identified 24 metabolites that differentiate between NW and OB patients. In particular, concentrations of pyroglutamic acid and propylamine were higher in OB patients compared to those of NW, while niacin and butanedioic acid were more abundant in NW patients (Figure 2). Interestingly, comparative orthology analyses of 12 *Finegoldia* genomes uncovered a sequence encoding a pyrrolidone-carboxylate peptidase in all the genomes.^22^ This enzyme catalyzes the removal of pyroglutamic acid from peptides and proteins,^32^ playing a role in the degradation of exogenous polypeptides or in the detoxification of N-terminally blocked peptides.^33^ These findings are in good agreement with our observation of elevated pyroglutamic acid levels in OB patients with colon polyps compared to those of NW, suggesting a possible correlation between the increased presence of *F. magna* in OB patients and higher pyroglutamic acid levels. However, integrated analysis failed to reveal any statistically significant correlation between the bacterial cluster that includes *Finegoldia* and any metabolite class of the mucosa-associated samples (Figure S9). Hence, it appears that the enrichments of *F. magna* and pyroglutamic acid in OB patients are not directly correlated. This metabolite has been found upregulated in the serum of patients with ulcerative colitis^34^ and in feces of subjects with irritable bowel disease.^35^ *In vitro* experiments showed that pyroglutamic acid treatment increased IL-6 mRNA level and DNA damage, suggesting that it can promote neoplastic transformation.^34^ Interestingly, pyroglutamic acid is a marker of glutathione deficiency and high oxidative stress, being an intermediate of glutathione metabolism where an increase in oxidative stress leads to a decrease in glutathione.^36^ Moreover, our integrated analysis of the mucosa-associated data showed a negative correlation between pyroglutamic acid and both *V. dispar* and *E. albertii* (Figure 4). *V. dispar* has been reported in infection cases,^37^ while *E. albertii* is recognized as an attaching-effacing pathogen causing lesions on the intestinal mucosa.^38^ The fact that these species did not appear in the LDA-LEfSe analysis of NW vs. OB patients suggests their potential role in pyroglutamic acid consumption in either group.

Niacin, also known as vitamin B3, nicotinamide, or nicotinic acid, can be obtained from the diet or synthesized from aspartic acid or tryptophan by the gut microbiota.^39^ Its active form, nicotinamide adenine dinucleotide (NAD), plays a critical role in various metabolic processes.^39^ Indeed, niacin deficiency can lead to pellagra, a disease characterized by inflamed skin, diarrhea and/or dementia.^40^ It has been reported that niacin not only reduces inflammation and oxidative stress but also increases adiponectin levels, an anti-inflammatory hormone, thereby mitigating inflammation and insulin-resistance in adipose tissue induced by a high-fat diet in mice.^41^ Moreover, niacin, as well as butyrate, can act as a ligand for the GPR109A receptor (also known as HCA2), located on intestinal epithelial and immune cells. GPR109A, encoded by the *Niacr1* gene, plays a crucial role in maintaining intestinal health. Mice deficient in Niacr1 (Niacr1^−/−^) exhibit decreased survival rates, experience significant body weight loss, and are more susceptible to colonic inflammation and colon cancer,^42^^,^^43^ highlighting the importance of this receptor in protecting against gastrointestinal diseases. This relationship is further underscored by observations that GPR109A expression is silenced in both mice and humans with colon cancer,^44^ indicating a potential therapeutic target for preventing or managing the disease.

Interestingly, *F. magna* is predicted to be auxotrophic for vitamin B3, thus unable to synthesize this nutrient.^45^ Therefore, it is tempting to speculate that *F. magna*, found in higher concentrations in MAM of OB patients, utilizes host-derived niacin, leading to its reduced availability in these individuals, who then become more susceptible to colonic inflammation and colon cancer. It is plausible that other bacteria may also use niacin, potentially affecting its levels. This hypothesis is supported by our integrated analysis between mucosa-associated microbiota and mucosa-associated metabolome, which failed to find a correlation between the bacterial cluster to which *Finegoldia* belong and any metabolite class (Figure S9). Hence, the observed changes in metabolite concentrations could stem from variations in bacterial consortia between OB and NW patients, rather than from the influence of a single bacterium like *F. magna*.

Unlike OB patients, the MAM and metabolome of NW patients were characterized by an enrichment of *Roseburia inulinivorans* and butanedioic acid (also known as succinic acid). Intriguingly, studies in mice have shown that adopting a diet low in plant polysaccharides and fibers leads to reduced luminal cecal concentrations of succinate and to a lower abundance of *R. inulinivorans*.^46^ Although the difference was not statistically significant, we observed a slightly higher fiber intake among NW patients compared to their OB counterparts. Collectively, these findings suggest an interplay among fiber consumption, higher abundance of *R. inulinivorans*, and butanedioic acid in NW patients with colorectal tumors.

Fecal metabolite alterations in CRC patients have been reported in a variety of researches,^47^ while in this study we mainly focused on obese patients with non-malignant colorectal polyps.

Higher levels of hexanedioic acid, also known as adipic acid, were found in fecal samples from obese patients. The molecule has been already linked to the development of colorectal and lung cancer.^48^

On the other hand, some metabolites that have been reported to be associated to CRC are less represented in our OB respect to NW patients. Thus, in obese patients with colorectal polyps these metabolites may have a less important role in carcinogenesis. For example, although many research proposed bile acids as strong carcinogens or promoters of colon cancers,^49^^,^^50^ we found lower levels of lithocholic acid in obese patients compared to NW. Furthermore, succinic acid, which has been considered an oncometabolite in colorectal cancer^51^ and was identified at higher concentration in stools from CRC patients,^52^ was downregulated in mucosal samples of obese patients compared to NW.

Similarly, a potential protective molecule, indol-5-ol, was higher in obese individuals. This molecule, also referred to 5-hydroxyindole (5HIAA), is a catabolite of serotonin, whose synthesis is induced in colon chromaffin cells by intestinal sporigenic microbes. Intriguingly, we have found two sporigenic genera enriched in the feces of obese individuals (Clostridium and Erysipelotrichaceae).^53^ High levels of 5-HIAA have been found in patients with metabolic syndrome, a condition that includes obesity and insulin resistance.^54^ This molecule is known for its anti-inflammatory properties and potential anti-cancer mechanisms.^55^

These data suggest that different factors influence colorectal carcinogenesis in obese and normal-weight individuals.

In conclusion, this study represents the first attempt to evaluate tumor-adherent microbiota and metabolome across a comprehensive cohort of OB and NW patients with colon polyps, complemented by an analysis of their diet and lifestyle habits through standardized questionnaires. Our data support the hypothesis that different risk factors may cooperate in tumorigenesis of OB and NW patients. We confirm the role of dietary risk factors, such as the consumption of processed meats in OB patients, and propose the involvement of the gut microbiota, exemplified by the enrichment of *F. magna*, and its metabolome in obese patients, in colorectal carcinogenesis. This is the first work to show an association between the abundance of *F. magna* and the presence of colorectal polyps in OB patients. Further functional studies are clearly needed to characterize the molecular mechanisms of this association.

### Limitations of the study

This study, while providing valuable insights into the interactions between obesity, diet, gut microbiota, and colorectal cancer risk, has several limitations. The cross-sectional design identifies associations but cannot establish causality, underscoring the need for longitudinal studies. We recruited only patients with polyps exceeding 1 cm in size at colonoscopy and, therefore, the polyp dimension was a selection bias of the study. Another limitation of this work is the use of the original LEfSe analysis that does not include FDR correction^56^ for microbiota analyses. LEfSe is, however, largely used and more sensitive than other methods.^57^ Biological tests will be necessary to confirm the role of the observed results. Dietary data, collected through standardized questionnaires, may be subject to recall bias. Thus, more objective measures could enhance accuracy. Lastly, our findings may not be extended to diverse populations with different dietary habits, highlighting the importance of research in multiple demographic settings.

## Resource availability

### Lead contact

Further information and requests for resources and reagents should be directed to and will be fulfilled by the lead contact, Irma Dianzani (irma.dianzani@med.uniupo.it).

### Materials availability

This study did not generate new unique reagents.

### Data and code availability

Raw 16S rRNA gene sequencing data has been deposited in Zenodo database accessed in March 2025 (mucosa-associated raw data: https://doi.org/10.5281/zenodo.14989507 and lumen-associated raw data: https://doi.org/10.5281/zenodo.14989583). Metabolome raw data (fecal data in sheet1, mucosal data in sheet2, volcano plots in sheet3) has been deposited in Zenodo database accessed in March 2025 (https://doi.org/10.5281/zenodo.14989816). Accession numbers are listed in the key resources table.

This paper does not report original code.

Any additional information required to reanalyze the data reported in this paper is available from the lead contact upon request.

## Acknowledgments

The research leading to these results has received funding from AIRC (Associazione Italiana per la Ricerca sul Cancro) under IG 2021-ID. 25886 project–P.I. Irma Dianzani, and from the 10.13039/501100003407Italian Ministry of Education, University and Research (MIUR) program “Departments of Excellence 2018–2022”, FOHN Project – Department of Health Sciences, and “Departments of Excellence 2023–2027”, AGING Project – Department of Translational Medicine, Università del Piemonte Orientale.

We sincerely thank Santina Castriciano (COPAN Italia SpA) for providing eNAT swabs to collect mucosal-adherent microbiota and Marcello Arsura (Abeschool) for reviewing the English language.

Graphical abstract was created with BioRender.com.

## Author contributions

M.L.V.: Investigation, formal analysis, data curation, visualization, and writing – original draft. M.G.C.: Investigation, formal analysis, data curation, and writing – original draft. M.S.: Investigation and writing – review and editing. G.S.: Investigation, visualization, and writing – review and editing. D.M.: Investigation, visualization, and writing – review and editing. E. Barberis: Investigation and writing – review and editing. S.J.: Resources and writing – review and editing. M. Mellai: Investigation and writing – review and editing. N.P.: Resources and writing – review and editing. R.B.: Resources and writing – review and editing. B.A.: Writing – review and editing. E. Bona: Writing – review and editing. E.P.: Formal analysis and writing – review and editing. F.P.: Writing – review and editing. C.S.: Writing – review and editing. D.F.: Formal analysis and writing – review and editing. E.G.: Writing – review and editing. M. Manfredi: Funding acquisition, formal analysis, visualization, and writing – original draft. A.A.: Supervision, project administration, conceptualization, visualization, and writing – original draft. I.D.: Supervision, funding acquisition, project administration, conceptualization, visualization, and writing – original draft.

## Declaration of interests

The authors declare no competing interests.

## STAR★Methods

### Key resources table

**Table:** 

REAGENT or RESOURCE	SOURCE	IDENTIFIER
**Biological samples**		

Stool samples	Patients with colorectal polyps	
DNA from swabs brushed on polyps	Patients with colorectal polyps, e-NAT™ swabs (COPAN, Brescia, Italy)	e-NAT™ swabs Cat# 608CS01R

**Critical commercial assays**		

QIAamp® DNA Microbiome kit	Qiagen, Hilden, Germany	Cat# 51704
QIAamp® PowerFecal® Pro DNA kit	Qiagen, Hilden, Germany	Cat# 51804
Microbiota Solution B Kit	Arrow Diagnostics Srl, Genoa, Italy	Cat# AD-002.024
MiSeq Reagent Nano Kit v2	Illumina, San Diego, CA, USA	Cat# MS-103-1003

**Deposited data**		

Mucosa-associated microbiota raw data	Mucosa-associated microbiota raw data related to the article "Gut microbiota and metabolome signatures in obese and normal-weight patients with colorectal tumors"	Zenodo: https://doi.org/10.5281/zenodo.14989507
Lumen-associated microbiota raw data	Lumen-associated microbiota raw data related to the article "Gut microbiota and metabolome signatures in obese and normal-weight patients with colorectal tumors"	Zenodo: https://doi.org/10.5281/zenodo.14989583
Metabolome raw data	Metabolome raw data related to the article "Gut microbiota and metabolome signatures in obese and normal-weight patients with colorectal tumors"	Zenodo: https://doi.org/10.5281/zenodo.14989816

**Software and algorithms**		

MicrobAT Suite v1.2.1	SmartSeq srl, Novara, Italy	
ChromaTOF v5.31	Leco Corp., St. Josef, MI, USA	
MicrobiomeAnalyst	https://www.microbiomeanalyst.ca/	
MetaboAnalyst	www.metaboanalyst.ca	
3MCor	http://3mcor.cn/	

### Experimental model and study participant details

#### Human participants

The study involved 120 incidental Italian patients with colorectal polyps, among which 11 presented malignant sections. Patients were recruited from June 2019 to December 2021 at the Gastroenterology Unit of University Hospital "Maggiore della Carità" in Novara, Italy. The study was approved by Ethical Committee of Maggiore della Carità Hospital (Novara, Italy. Study number CE78/19; 7/6/2019) in accordance with the current legislation and the Declaration of Helsinki. Prior to undergoing colonoscopy, each participant provided written informed consent. Inclusion criteria mandated the presence of polyps exceeding 1 cm in size and an age threshold of 18 years or older. Clinical features of the patients are reported in Table 1. In particular, 70 males and 50 females of European ethnicity have been recruited. Sex does not influence the results of the study, as reported in Table 1 (*p* = 0.39).

Anthropometric parameters including height, weight, and waist circumference, were collected 14 days after colonoscopy and were used to classify patients in two different groups based on body mass index (BMI) and waist circumference (WC), according to World Health Organization cut-off points (Geneva, 8–11 December 2008). Subjects and derived samples were allocated to normal-weight (NW) or overweight/obese (OB) experimental groups. Individuals were classified as normal weight (NW) if they had a BMI <25 kg/m^2^ and WC ≤ 88 cm for women and 102 cm for men. Conversely, subjects were categorized as overweight/obese (OB) subjects with BMI ≥25 kg/m^2^ and/or WC > 88 cm for women and 102 cm for men.

### Method details

#### Sample collection

MAM was carefully collected using an e-NAT (COPAN, Brescia, Italy) swab, which was gently brushed on the adenoma surface without compromising the tissue integrity,^16^ before being stored at −80°C. To analyze LAM, fecal samples were collected from patients 14 days post-colonoscopy. These samples were then aliquoted and preserved at −80°C for subsequent analysis.

#### Histology

Polyps were preserved in neutral buffered formalin and embedded in and embedded in paraffin before being sectioned into 4-μm slices and stained using hematoxylin-eosin (H&E). A pathologist at the University Hospital in Novara, Italy, conducted a comprehensive evaluation of all polyps. For the purpose of categorization based on anatomical location, polyps were classified as proximal, comprising the cecum, ascending colon, hepatic flexure, and transverse colon, or distal, encompassing the splenic flexure, descending sigmoid, and rectal colon.^58^

Based on polyp histology, patients with hyperplastic polyps, serrated polyps without dysplasia, or low-grade dysplasia adenomas were grouped under the “low-grade” category, while patients with high-grade dysplasia adenomas, high-grade dysplasia serrated polyps or adenocarcinomas were assigned to the “high-grade” group (Table 1). Hyperplastic polyps were included in the “low-grade” group because hyperplasia represents the first step in multistep carcinogenesis.^59^ Adenocarcinomas were included in the “high grade” group because they are a portion of the polyps with early-stage malignant transformation. When we refer to “low-grade” or “high grade” group, all the polyps are included in the analyses. We also performed a refined microbiota analysis by excluding patients with hyperplastic polyps, serrated polyps, and adenocarcinomas.

#### Mucosa-associated and lumen-associated microbiota analyses

Microbial DNA was extracted from MAM collected by e-NAT swabs and from LAM in fecal samples using the QIAamp DNA Microbiome kit and the QIAamp PowerFecal Pro DNA kit (Qiagen, Hilden, Germany), respectively, according to the manufacturer’s instructions.

For both MAM and LAM, 16S rRNA amplicon sequencing analysis was performed using Microbiota Solution B Kit (cod. AD-002.024), which is CE-IVD marked (Arrow Diagnostics Srl, Genoa, Italy). Degenerate primer sets targeted the V3-V4-V6 hypervariable regions of bacterial 16S rRNA gene were used for amplification by polymerase chain reaction (PCR). The PCR products were then purified with Agencourt AMPure XP magnetic beads (Beckman Coulter Inc., Brea, CA, USA). After fluorometric quantification, the PCR libraries were pooled in equimolar concentrations. The sequencing of these amplicon libraries was performed on a MiSeq Illumina sequencing platform (Illumina, San Diego, CA, USA) using a MiSeq Reagent Nano Kit v2 cartridge for a 2x250 paired-end sequencing.

#### Raw sequence processing

MAM and LAM raw sequences were processed using the software MicrobAT Suite v1.2.1 (SmartSeq srl, Novara, Italy), based on the Ribosomal Database Project (RDP) database (v11.5). Sequences with a read length < than 200 nt or with a low quality (average Phred quality score^60^ < than 25) were removed. Sequences aligned to the reference database^61^ were used to generate three files (OTU, taxonomy, metadata) that represent the input for the statistical analyses.^62^^,^^63^ Statistical analysis was performed using MicrobiomeAnalyst software v2 (Comprehensive Statistical, Visual, and Meta-Analysis of Microbiome data)^64^ that removed all the taxa having zero reads across all the samples or appearing in only one sample and applied a low-count filter to remove taxa with less than 4 reads in at least 20% of samples.

#### Metabolome analysis

Metabolomic analysis was performed using both targeted and untargeted approaches. Short chain fatty acids (SCFAs) were quantified through a targeted approach, while remaining metabolites, including amino acids, organic acids, sugars, medium and long chain fatty acids, and many more were quantified using an untargeted approach.

Mucosa-associated metabolome was collected brushing a dry swab on the polyp surface of 115 patients (5 samples were unavailable). Small molecules were extracted and analyzed as previously reported.^16^^,^^65^ Briefly, SCFAs extraction was performed using water and sonication and then liquid-liquid extraction with methyl *tert*-butyl ether, while other metabolites were extracted from the aqueous phase using methanol-isopropanol-acetonitrile.

Feces aliquots of 79 patients were available for luminal metabolome analyses. For fecal metabolome analysis, approximately 30 mg of feces were placed in a tube, then 300 mL of water and 15 μL of propanoic acid d2 (20.4 ppm) and 15 μL of acetic acid d4 (0.1 mg/mL) were added as internal standards. The mixture was homogenized with the tissue lyser for three cycles of 40 s at a speed of 6.5 m/s. The tube was then incubated for 30 min at 4°C and 1000 rpm and then centrifuged for 30 min at 4°C and 21100x g. For SCFAs extraction, 140 μL of methyl *tert*-butyl ether (MTBE) were added to 100 μL of water-extract, the sample was mixed for 15 min at 40 rpm and then centrifuged for 10 min at 4°C and 21100x g.

A second extraction was performed on the remaining water phase solution. Briefly, small molecules were extracted from 100 μL of the sample using a 1 mL mixture of acetonitrile (ACN), isopropanol (IPA), and water 3:3:2, with 5 μL of tridecanoic acid (0.5 mg/mL), 5 μL of palmitic acid d31 (0.5 mg/mL), 5 μL of stearic acid d35 (0.5 mg/mL), 3.5 μL of glycine d4 (10.07 mg/mL) as internal standards. After being vortexed, the sample was centrifuged for 15 min at 20 °C at 14500x g. Next, 1 mL of the supernatant was dried in a speed vacuum at 40°C and stored at −20°C until derivatization, which involved two steps: methoximation (20 μL of methoxamine at 80°C for 20 min) and silylation (30 μL of N,O-Bis(trimethylsilyl)trifluoroacetamide at 80°C for 20 min).

SCFAs and small molecules from both swab and fecal samples were analyzed by bidimensional gas chromatography-mass spectrometry (GCXGC/TOFMS, BT 4D, Leco Corp., St. Josef, MI, USA), employing both targeted and untargeted approaches as described in our previous works.^16^^,^^65^ Raw data were processed by ChromaTOF (version 5.31), with mass spectral assignment achieved by matching against the NIST MS Search 2.3 libraries and the Fiehn Library. The identification of molecules was also performed using an in-house library comprised of commercial mix standards containing hundreds of molecules.

#### Microbiota-metabolome integrated analysis

To evaluate potential correlations between polyp-associated microbiota and its metabolites, the metabolome analysis was integrated with MAM data using 3MCor (http://3mcor.cn/, accessed in February 2024), an open-source web server. Hierarchical clustering and heatmap analysis were performed through MetaboAnalyst software 5.0 (www.metaboanalyst.ca, accessed in February 2024) using the Euclidean distance as distance measure and the Ward method for clustering. The criteria for inclusion in this analysis were strictly defined. Only metabolites demonstrating significant modulation (*p* < 0.05 and fold change (FC) > 1.3 or <0.769) were considered. We focused our integration analysis only on mucosa-associated samples because we thought that we could obtain relevant parameters for colorectal carcinogenesis.

#### Dietary habits

Dietary habits were evaluated using the validated European Prospective Investigation into Cancer and nutrition (EPIC) questionnaire^15^ for 119 patients. This comprehensive questionnaire consists of 16 categories and includes questions covering 266 food items. Statistical analysis of the questionnaire data enables the conversion of frequency intake for various nutrients into grams per day.

Using the results from this questionnaire, we conducted a comparison of the consumption levels of key nutrients between NW and OB patients, specifically focusing on red meat, processed meat, fruits and vegetables, total fiber, and total lipids. Moreover, the following nutrients consumptions were considered to calculate the adherence index to Mediterranean diet: pasta, vegetables, fruit, legumes, olive oil, alcohol, soft drinks, red meat, fish, potatoes, and butter. The adherence index to Mediterranean diet was calculated as previously reported.^66^

In addition to the EPIC questionnaire, we asked whether the patients had taken antibiotics or probiotics in the six months prior to colonoscopy.

#### Lifestyle habits

Lifestyle habits (smoking and physical activity) and educational level were evaluated using the validated European Prospective Investigation into Cancer and nutrition (EPIC) questionnaire^15^ for 120 patients. The physical activity index was calculated as previously reported.^67^

### Quantification and statistical analysis

#### Statistical analyses

The chi-squared test was used to assess the differences in sex, polyp localization, dysplasia grade, smoking and comorbidities between NW and OB patients. The unpaired t-test was used to determine differences between the age, the polyp dimension, the BMI and the WC of the different groups of patients. The Fisher’s exact test was used to assess the differences in polyp histology, physical activity, previous gastrointestinal conditions and educational level. A *p*-value <0.05 was considered statistically significant.

In analyzing the microbiota, heat tree analysis allowed us to compare statistically significant differences between the two groups (NW vs. OB). This analysis leverages a hierarchical taxonomy structure to quantitatively—through median abundance—and statistically—using non-parametric Wilcoxon Rank Sum test—depict taxon differences among communities.^68^

The observed number, the Shannon index, and the Simpson index were used to measure the α-diversity in NW and OB groups, and the statistical analysis was performed using Mann-Whitney test. β-diversity was analyzed using Bray-Curtis index as distance method and principal coordinate analysis (PCoA) as ordination method, and the statistical analysis was performed using permutational ANOVA (PERMANOVA).

The linear discriminant analysis effect size (LDA-LEfSe) methodology estimates both statistical significance and biological consistency (i.e., effect size). Thus, it was employed to identify statistically enriched taxa characterizing each group (NW vs. OB). LEfSe uses the Kruskal-Wallis sum-rank test to identify taxa statistically different between groups, then applies LDA to calculate the effect size of the different abundant taxa. Considering a power equal to 0.8, a type I error = 0.05, with 75 normal-weight and 45 obese patients (total 120 patients), the effect size was d = 0.5 (difference between two independent means of relative abundance in a species) according to Cohen’s classification. A medium effect size (d = 0.5) suggests a meaningful difference in microbiota composition that is likely to have biological relevance. Taxa were considered discriminative between groups if they had an *p* < 0.05 (original) and an LDA score >2 for enrichment in the first group relative to the second one, or < - 2 for enrichment in the second group relative to the first one.

For the metabolome analyses, partial least square discriminant analysis (PLS-DA) was applied to identify metabolites significantly relevant to each group, as described by Barberis et al..^65^ An FC > 1.3 was indicative of enrichment in the OB group, while an FC < 0.769 indicated a downregulation in the same group.

Correlations between microbiota and metabolites were assessed by Spearman’s rank correlation coefficient using a 3MCor web server, with significance set at *p* < 0.05.

The Mann-Whitney test was used to determine differences in food categories consumption between OB vs. NW patients (Table 2), between low-grade vs. high-grade groups (Table 3), and between the patients carrying *Finegoldia* on their polyps vs. the patients who did not. Food group and nutrient intakes were adjusted for total energy intake by the regression-residual method based on the distribution of residuals.^69^ Correlations between levels of *Finegoldia* and levels of processed meat consumption were assessed by Spearman’s rank correlation coefficient. A *p*-value <0.05 was considered statistically significant.

## Supplemental information

Document S1. Figure S1−S9 and Table S1
