# Supplementary material for: Gut microbiota and metabolome signatures in obese and normal-weight patients with colorectal tumors
Source: iScience. 2025 Mar 13;28(4):112221. doi: 10.1016/j.isci.2025.112221 (PMC11995084; doi:10.1016/j.isci.2025.112221)
Supplement: Document S1. Figure S1−S9 and Table S1 [file mmc1.pdf]

iScience, Volume *28*

Supplemental information

Gut microbiota and metabolome signatures in obese and normal-weight patients with colorectal tumors

Marta La Vecchia, Michela Giulia Clavenna, Marika Sculco, Gloria Sala, Denise Marradi, Elettra Barberis, Soni Joseph, Marta Mellai, Nico Pagano, Renzo Boldorini, Barbara Azzimonti, Elisa Bona, Edoardo Pasolli, Flavia Prodam, Carlotta Sacerdote, Daniela Ferrante, Emilia Ghelardi, Marcello Manfredi, Anna Aspesi, and Irma Dianzani

Supplemental Information

Table of contents

Figure S1. Flow chart of the analysis performed on the study population……………………..**pag. 3**

Figure S2. α-diversity and β-diversity analyses for MAM samples……………………………**pag. 4**

Figure S3. Bacterial species enriched in MAM of NW vs OB patients considering only patients with tubular, tubulovillous or villous polyps……………………………...………………………..**pag. 5**

Figure S4. Bacterial species enriched in MAM of the obese patients when categorized by dysplasia grade of their polyps……………………………………………………………………**pag. 6**

Figure S5. Bacterial genera enriched in LAM from NW patients vs OB patients……………..**pag. 7**

Figure S6. Bacterial genera enriched in LAM of NW vs OB patients considering only patients with tubular, tubulovillous or villous polyps……………………………...………………………**pag. 8**

Figure S7. Bacterial species enriched in LAM of the obese patients when categorized by dysplasia grade of their polyps……………………………………………………………………**pag. 9**

Figure S8. Box plots of some of the most statistically significant luminal metabolites in discriminating between NW and OB patients……………………………………………..**pag. 10**

Figure S9. Hierarchical clustering heatmap illustrating the correlation between groups of metabolites and groups of microbes…………………………………………………………………...**pag. 11**

Table S1. Enrichment of *F. magna* or *unclassified Finegoldia* in subgroups of patients……..**pag. 12**

Figure S1

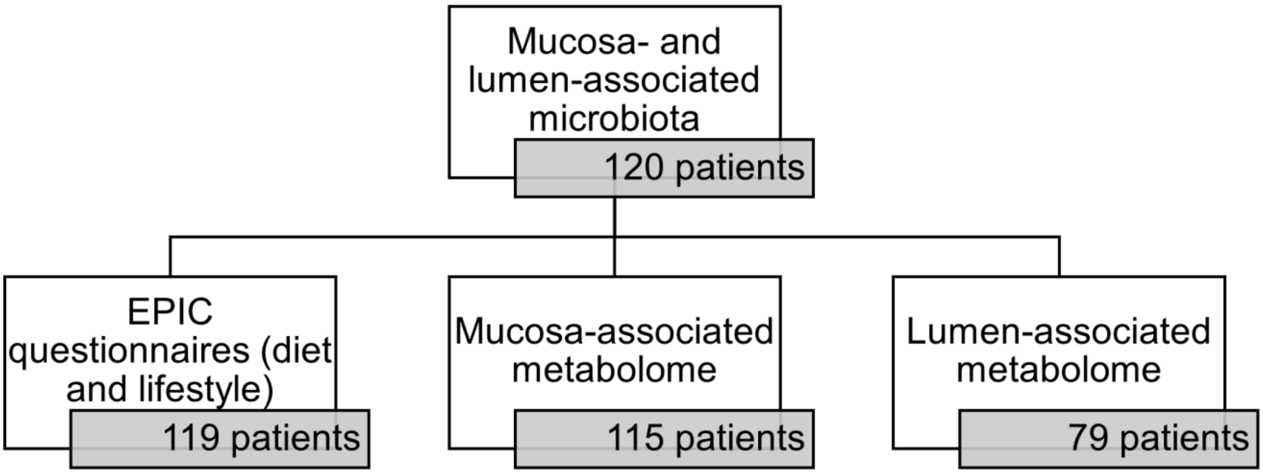

Figure S1. Flow chart of the analysis performed on the population involved in the study.

Figure S2

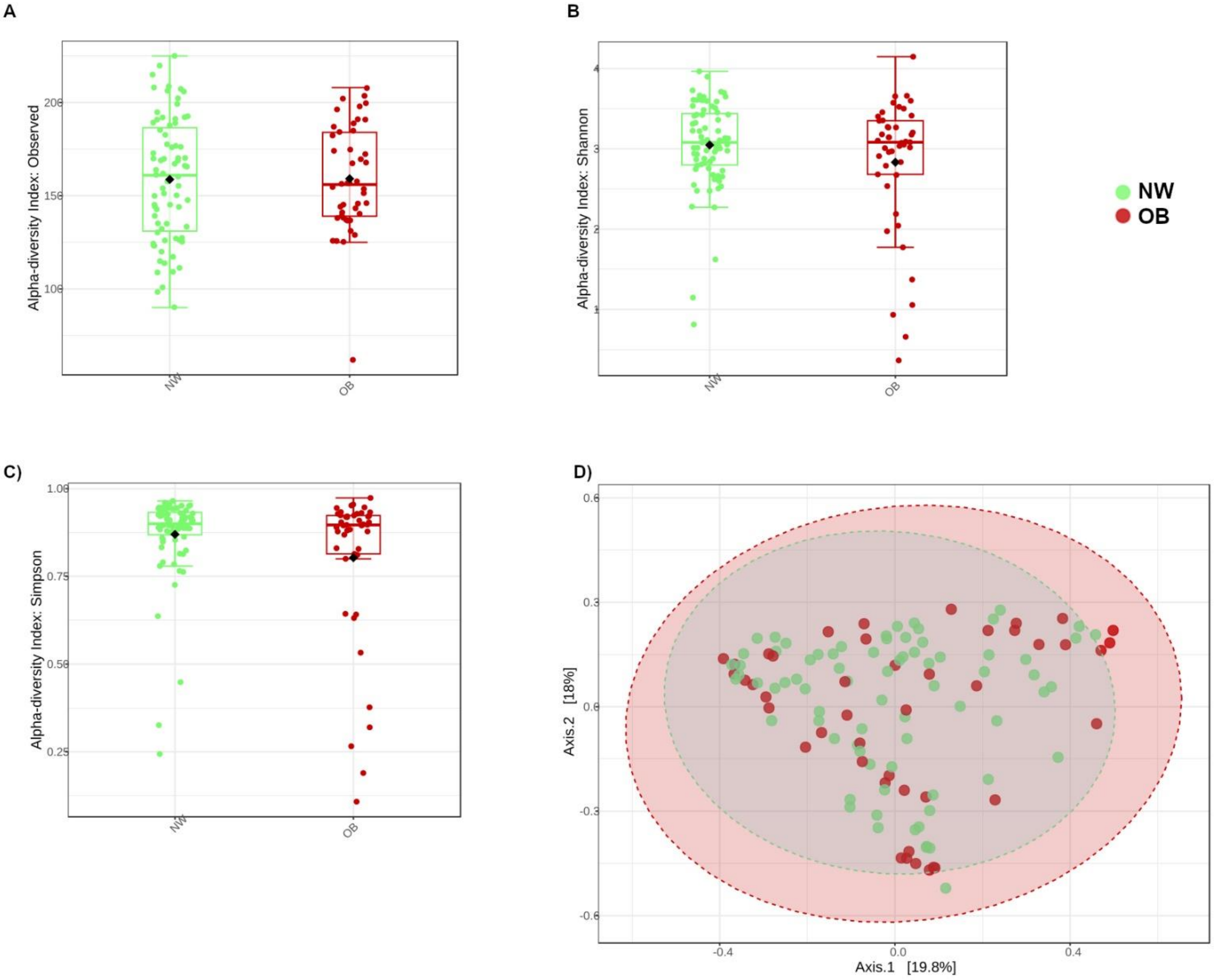

Figure S2. α-diversity and β-diversity analyses for MAM samples. (A, B, C) Box plots showing three different α-diversity indexes tested by Wilcoxon rank sum test. The box plots show the difference in the observed index (A), Shannon index (B) and Simpson index (C) between NW (green) vs OB patients (red) (observed: $p = 0.84$, Shannon: $p = 0.46$, Simpson: $p = 0.24$). (D) β-diversity analysis between the two group of patients ($p = 0.42$): principal coordinates analysis (PCoA), based on Bray–Curtis distance matrix, shows the different microbial composition between the two groups. The X-axis explains 19.8% of the variability between samples, while the Y-axis explains 18%. NW: normal-weight, OB: obese patients.

Figure S3

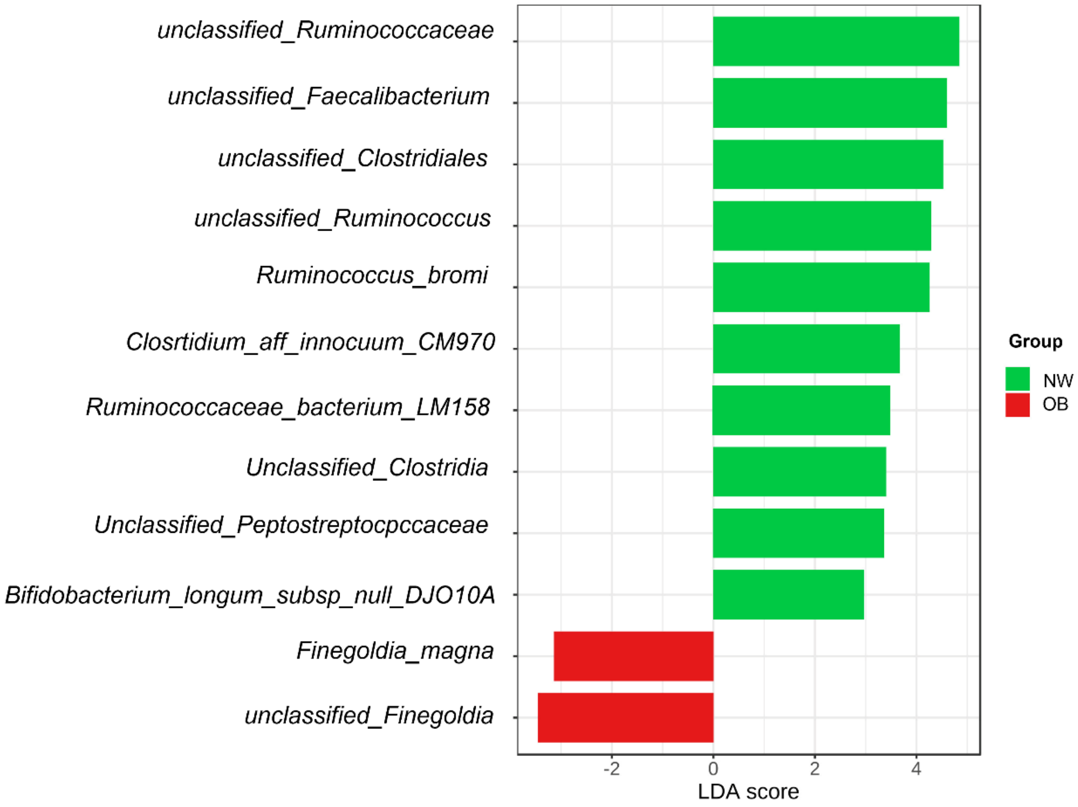

Figure S3. Bacterial species enriched in MAM of NW (green, LDA score > 2) vs OB patients (red, LDA score < −2) considering only patients with tubular, tubulovillous or villous polyps. Patients with hyperplastic polyps, serrated polyps, and adenocarcinomas were excluded from this analysis. The results show that *F. magna* enrichment in OB patients is independent of polyp histology. Data are represented as bars, ranking discriminative features found by LEfSe analysis ($p < 0.05$) according to their LDA score. NW: normal-weight, OB: obese patients.

Figure S4

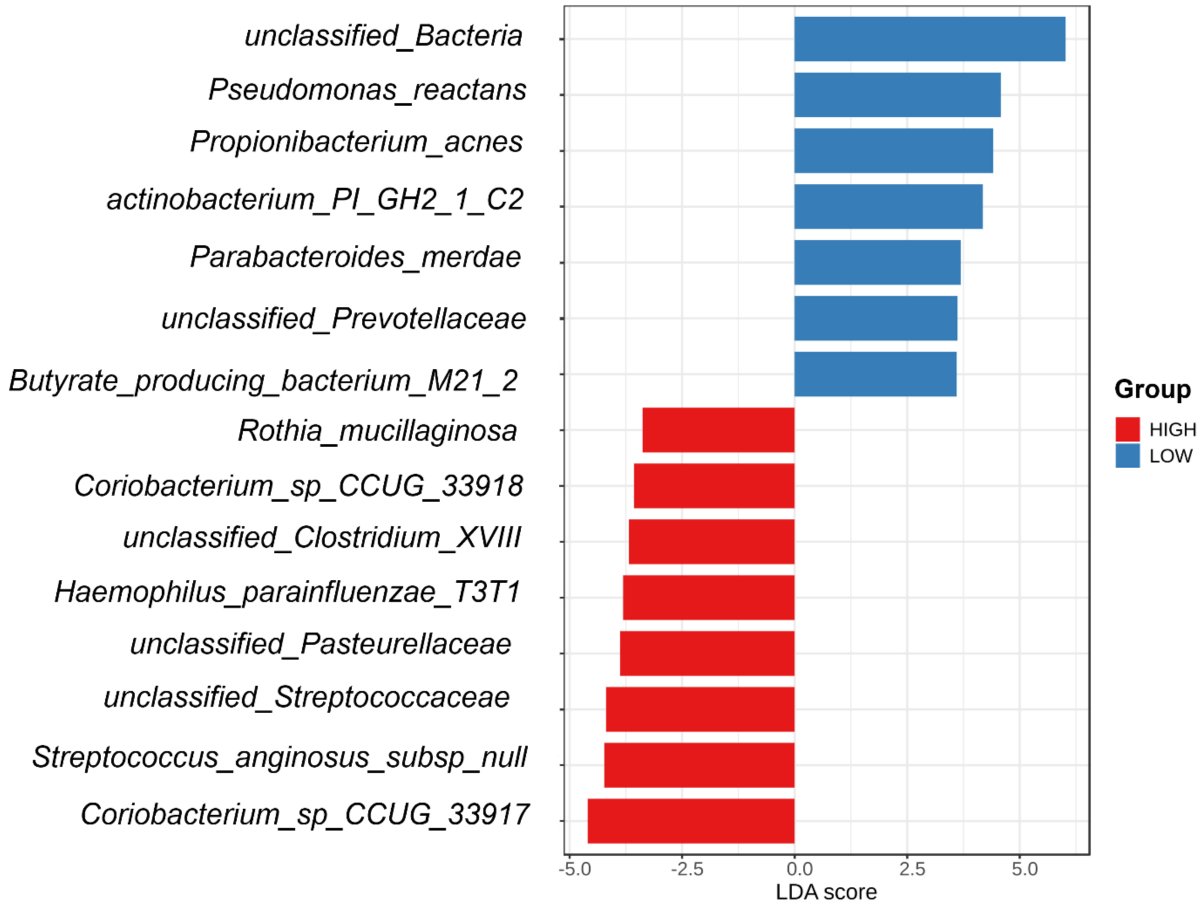

Figure S4. Bacterial species enriched in MAM of the obese patients when categorized by dysplasia grade of their polyps (high-grade, red, LDA score > 2 vs low-grade, blue, LDA score < −2). This analysis shows that *F. magna* enrichment in MAM from OB patients does not vary with the dysplasia grade of the colon polyps. Data are represented as bars, ranking discriminative features found by LEfSe analysis ($p < 0.05$) according to their LDA score.

Figure S5

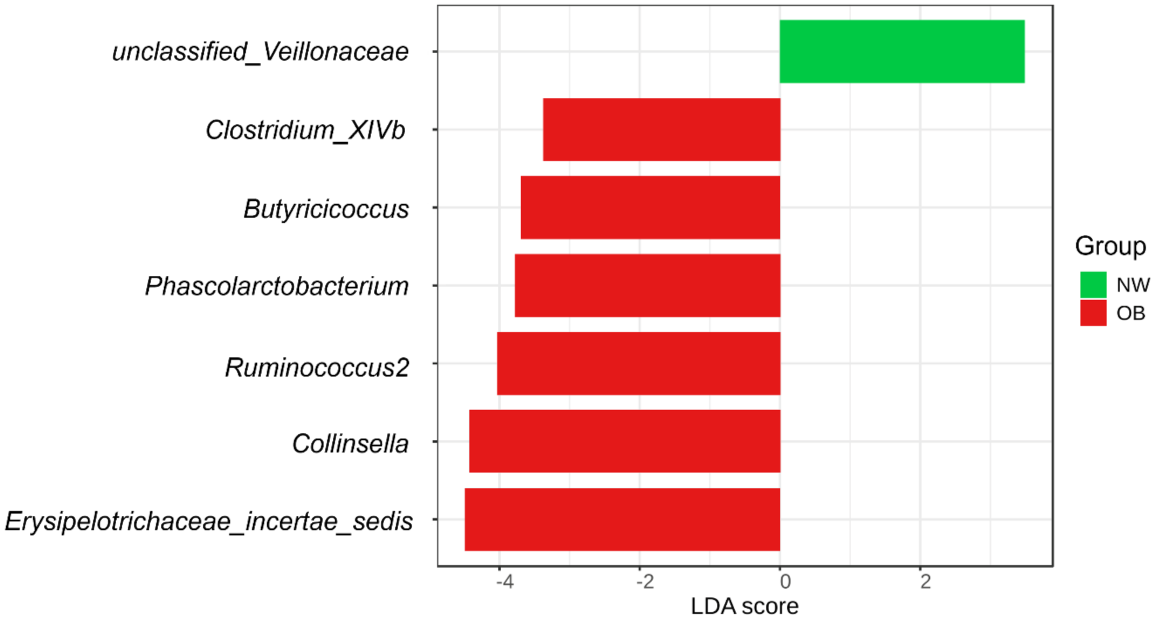

Figure S5. Bacterial genera enriched in LAM from NW patients (green, LDA score > 2) vs OB patients (red, LDA score < −2). Data are represented as bars, ranking discriminative features found by LEfSe analysis ($p < 0.05$) according to their LDA score. NW: normal-weight, OB: obese patients.

Figure S6

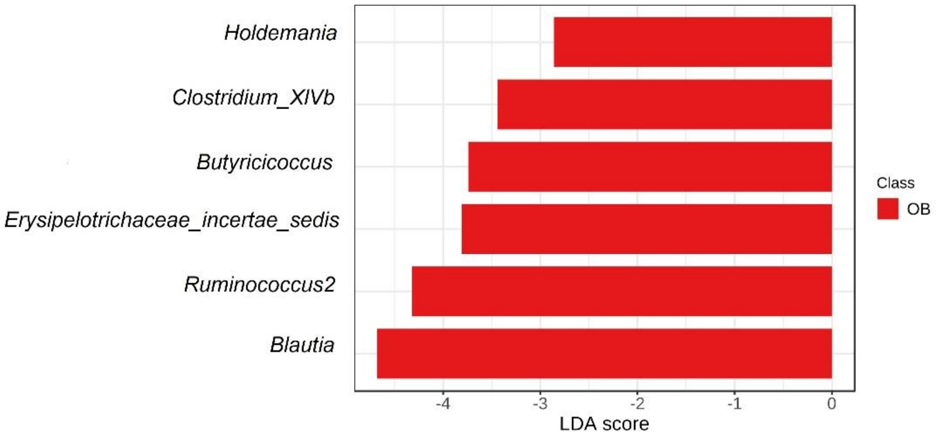

Figure S6. Bacterial genera enriched in LAM of NW (green, LDA score > 2) vs OB patients (red, LDA score < −2) considering only patients with tubular, tubulovillous or villous polyps. Patients with hyperplastic polyps, serrated polyps, and adenocarcinomas were excluded from this analysis. The results show that *Clostridium XIVb, Butyricicoccus, Erysipelotrichaceae incertae sedis, Ruminococcus2* enrichment in OB patients is independent of polyp histology. Data are represented as bars, ranking discriminative features found by LEfSe analysis ($p < 0.05$) according to their LDA score. NW: normal-weight, OB: obese patients.

Figure S7

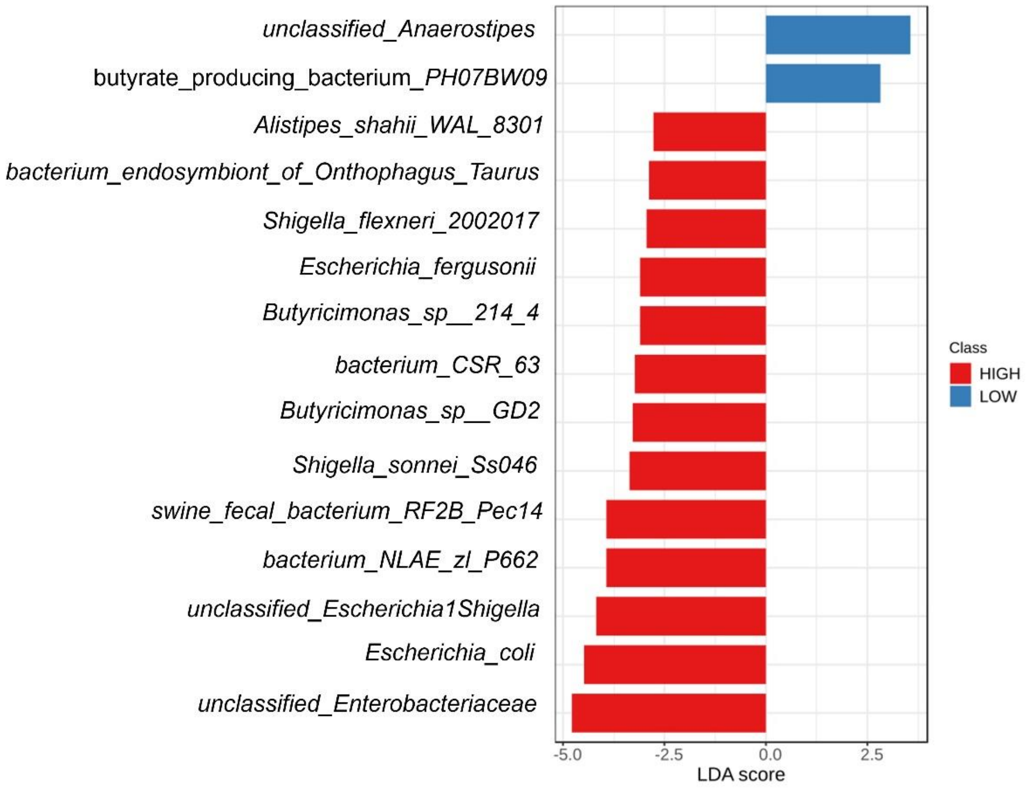

Figure S7. Bacterial species enriched in LAM of the obese patients when categorized by dysplasia grade of their polyps (high-grade, red, LDA score > 2 vs low-grade, blue, LDA score < −2). This analysis shows that none of the bacteria found enriched in LAM of OB patients when compared to NW vary with the dysplasia grade of the colon polyps. Data are represented as bars, ranking discriminative features found by LEfSe analysis ($p < 0.05$) according to their LDA score.

Figure S8

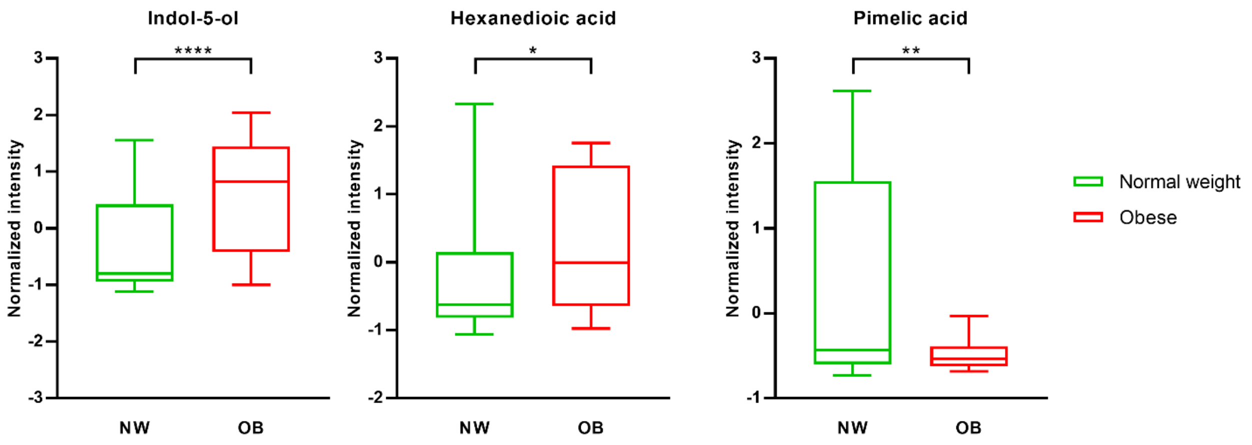

Figure S8. Box plots of some of the most statistically significant luminal metabolites in discriminating between normal-weight (NW, green) and obese (OB, red) patients (****: $p < 0.0001$; **: $p < 0.01$; *: $p < 0.05$). Data are represented as metabolite abundances.

Figure S9

A)

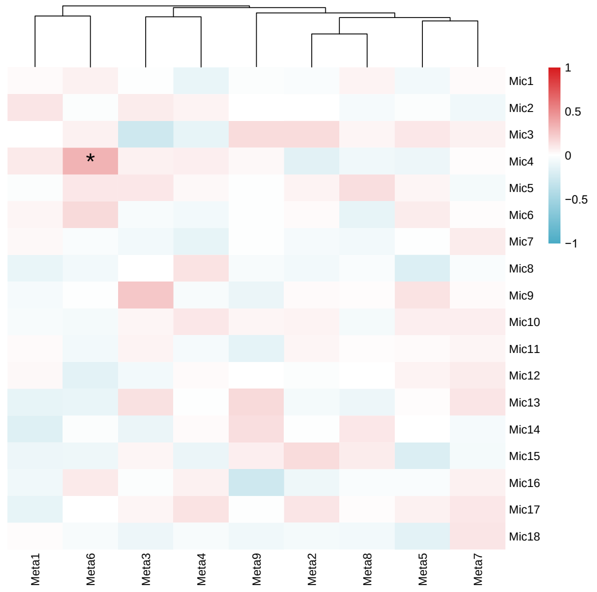

Mic4 cluster: *Microbacterium, Fusobacterium, Prevotella, Odoribacter, Phyllobacterium, Lachnospiracea incertae sedis, Clostridium XVII, Pelomonas, Alistipes, Blautia,* and *Escherichia Shigella*.

Meta6 cluster: hydracrylic acid, L-rhamnose, butanedioic acid, benzaldehyde, benzoic acid, 2H-pyranmethanol, linoleic acid, hexadecanal, pentadecanal, and benzeneacetic acid.

B)

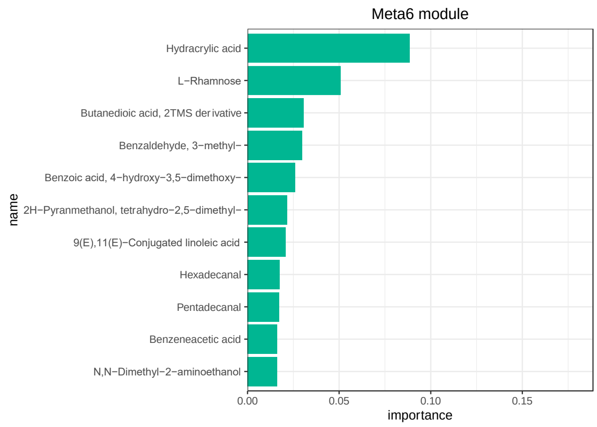

C)

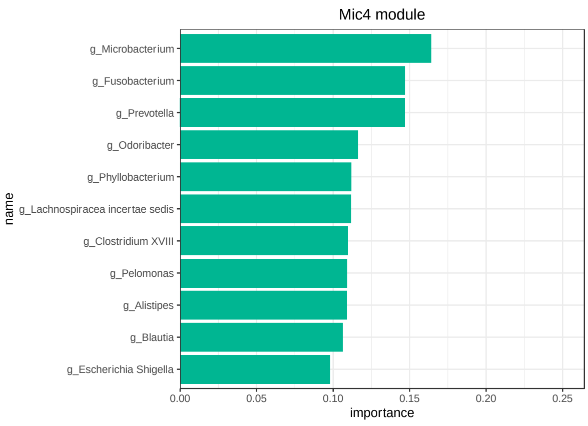

D)

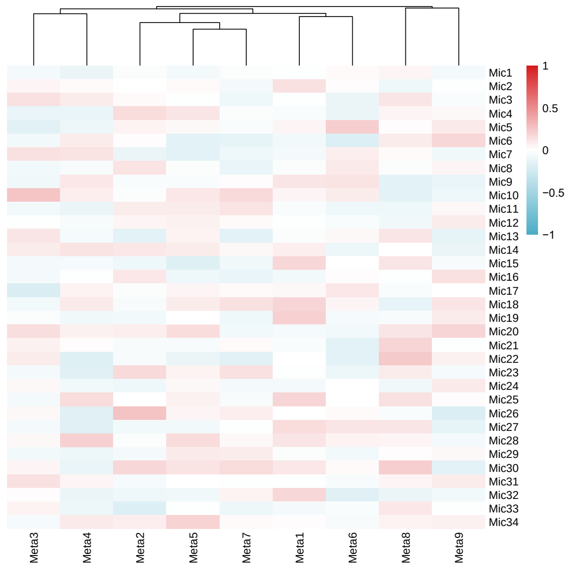

Figure S9. Hierarchical clustering heatmap illustrating the correlation between groups of metabolites and groups of microbes, both at the genus (A) and species (D) level. The only statistically significant correlation, marked with an asterisk in (A), is between Meta6 (B) and Mic4 (C) clusters.

Table S1. Enrichment of *F. magna* or *unclassified Finegoldia* in subgroups of patients.
The analyses are performed with Linear discriminant analysis effect size (LDA-LEfSe) on MAM ($p < 0.05$; LDA score > 2 or < −2).

**Subgroups**	**OB vs NW comparison in each subgroup (LEfSe)**
Distal polyp localization (n=75)	**Enrichment** of *unclassified Finegoldia* and *F.magna* in OB (n=30) vs NW (n=45) patients with distal polyps
Proximal polyp localization (n=45)	No enrichment of *unclassified Finegoldia* and *F.magna* in OB (n=15) vs NW (n=30) patients with proximal polyps
Adenomas (n=93)	**Enrichment** of *unclassified Finegoldia* and *F.magna* in OB (n=40) vs NW (n=53) patients with adenomas
Serrated pathway polyps (n=16)	No enrichment of *unclassified Finegoldia* and *F.magna* in OB (n=3) vs NW (n=13) patients with serrated pathway polyps
1st tertile adherence index to the Mediterranean diet (n=41)	No enrichment of *unclassified Finegoldia* and *F.magna* in OB (n=18) vs NW (n=23) patients
2nd tertile adherence index to the Mediterranean diet (n=61)	No enrichment of *unclassified Finegoldia* and *F.magna* in OB (n=23) vs NW (n=38) patients
3rd tertile adherence index to the Mediterranean diet (n=17)	No enrichment of *unclassified Finegoldia* and *F.magna* in OB (n=4) vs NW (n=13) patients
